# Relationship between red cell distribution width-to-albumin ratio and major adverse cardiovascular events among individuals with acute myocardial infarction

**DOI:** 10.3389/fcvm.2025.1663408

**Published:** 2025-09-25

**Authors:** Cai Chen, Xiaoguo Hua, Rui Hu, Jun Wan, Ya You, Min Yang

**Affiliations:** ^1^ Department of Emergency, The Second Affiliated Hospital of Anhui Medical University, Hefei, China; ^2^ Office of Medical Insurance Management, The Second Affiliated Hospital, Anhui Medical University, Hefei, China; ^3^ Department of Clinical Teaching Management, The First Affiliated Hospital, Anhui University of Traditional Chinese Medicine, Hefei, China; ^4^ Department of Cardiology, The Second Affiliated Hospital of Anhui Medical University, Hefei, China; ^5^ The Second Department of Intensive Care Unit, The Second Affiliated Hospital of Anhui Medical University, Hefei, China

**Keywords:** red cell distribution width-to-albumin ratio, major adverse cardiovascular events, acute myocardial infarction, composite index, biomarker

## Abstract

**Background:**

Red cell distribution width (RDW) and albumin have been individually linked to adverse cardiovascular outcomes, but the prognostic value of their composite index, the red cell distribution width-to-albumin ratio (RAR), in AMI patients remains under-explored.

**Methods:**

A retrospective cohort study was conducted on 917 AMI patients. The main outcome was in-hospital MACEs, including 28-day death, non-fatal stroke, recurrent myocardial infarction, unplanned revascularization, and hospitalization for heart failure or angina. Multi-variable logistic regression analysis was used to calculate their odd ratio (OR) and corresponding confidence interval (95%CI) with adjustments that assessed the relationship RAR index (categorical or continuous variable) of with MACEs among individuals with AMI. Restricted cubic spline (RCS) models were used to assess the dose-response relationship between RAR index and the incidence of MACEs. In addition, receiver operating characteristic curve (ROC) analysis was fitted to assess the accuracy of RAR index in predicting MACEs.

**Results:**

The cohort had a mean age of 58.7 ± 9.6 years, with 56% males. Higher RAR quartiles were associated with older age, higher MACEs incidence, and lower BMI, cholesterol, eGFR, and blood pressure (all *P* < 0.05). After full adjustment, each 1-unit increase in RAR was independently associated with elevated MACEs risk (OR = 1.34, 95% CI: 1.03–1.74, *P* = 0.030), and the highest RAR quartile (Q4) had a 2.30-fold higher risk than the lowest (Q1, *P* = 0.005). RCS analysis revealed a non-linear relationship with a critical RAR value of 3.48; above this threshold, MACEs risk increased significantly (OR = 2.17, *P* = 0.031). RAR showed superior predictive performance (AUC = 0.614) compared to RDW (0.564) or albumin (0.605). Subgroup analyses indicated significant associations in male patients (OR = 1.57, *P* = 0.002) and those with ST-segment elevation myocardial infarction (STEMI, OR = 1.32, *P* = 0.045).

**Conclusion:**

The predictive value of RAR surpasses that of RDW or albumin alone and varies by sex and AMI subtype. RAR holds promise as a simple, cost-effective biomarker for risk stratification in AMI patients, warranting further validation in prospective studies.

## Introduction

Acute myocardial infarction (AMI) is a leading cause of mortality and morbidity worldwide, imposing a significant burden on healthcare systems. Despite advances in treatment modalities, in-hospital complications and mortality remain high, underscoring the need for robust prognostic markers to identify high-risk patients early. For instance, a study reported that the mortality rate for AMI patients can be as high as 10% within 30 days of hospital admission, highlighting the critical need for better risk stratification tools (1). Current prognostic tools have limitations in accurately predicting outcomes, necessitating the exploration of novel biomarkers to improve risk stratification. Variability in clinical outcomes across different regions and healthcare settings further complicates the management of AMI patients. For example, a comparative analysis showed that in-hospital mortality rates for AMI can vary significantly, ranging from 4% in some regions to over 12% in others, depending on the availability of advanced medical facilities and the quality of care provided (2). This variability underscores the importance of identifying new prognostic markers that can provide more accurate and reliable predictions, potentially improving clinical decision-making and patient outcomes.

Red cell distribution width (RDW) has emerged as a promising biomarker in various cardiovascular conditions, reflecting underlying inflammatory and oxidative stress processes. Elevated RDW levels have been associated with adverse outcomes in patients with acute coronary syndromes, including increased mortality and risk of complications. For instance, a study reported that RDW levels above 14.5% were associated with a 2.5-fold increase in mortality risk among patients with acute myocardial infarction (3). Albumin, a key protein in the bloodstream, is often used as a marker of nutritional status and overall health. Low serum albumin levels have been linked to poor prognosis in cardiovascular diseases, potentially indicating chronic inflammation and malnutrition. A meta-analysis found that each 1 g/L decrease in serum albumin was associated with a 1.2-fold increase in mortality risk in patients with heart failure (4). While RDW and albumin have been studied individually, their combined potential as a prognostic marker in AMI has not been fully explored. This gap in knowledge highlights the need for further research to evaluate the combined predictive value of RDW and albumin in the context of AMI prognosis.

The red cell distribution width to albumin ratio RAR represents a novel composite biomarker that integrates the inflammatory and nutritional aspects reflected by RDW and albumin, respectively. This study aims to assess the prognostic significance of RAR in predicting in-hospital outcomes, including mortality, complications, and length of stay in patients with acute myocardial infarction. We hypothesize that RAR, by combining the complementary information from RDW and albumin, may offer enhanced risk stratification compared to either biomarker alone. For example, preliminary data from a retrospective analysis of 500 AMI patients showed that RAR values above 0.5 were associated with a 3.2-fold increase in in-hospital mortality compared to lower RAR values (1). Additionally, this study will explore whether RAR provides incremental value over established prognostic markers in AMI, potentially improving clinical decision-making and patient management. By integrating RDW and albumin, RAR may provide a more comprehensive assessment of patient risk, aiding in the identification of high-risk patients who may benefit from more aggressive interventions.

## Materials and methods

### Study design and participants

This study was a retrospective cohort analysis conducted on patients diagnosed with AMI who were admitted to the Second Affiliated Hospital of Anhui Medical University, China, between 2013 and 2023. The Ethics Review Committee at the Second Affiliated Hospital of Anhui Medical University has granted approval for the research protocol in this study (Approval No. SL-YX2025-153). Participants were included if they had a confirmed diagnosis of AMI based on clinical symptoms, electrocardiogram (ECG) findings, and elevated cardiac biomarkers. Patients were excluded from the analysis if they had missing data on RDW, albumin, or other covariates. Additionally, patients with a history of chronic liver disease, chronic kidney disease (stage 5), age below 18 years, or those who had received blood transfusions within 48 hours before blood sampling were excluded to minimize confounding factors that could affect RDW and albumin levels. A total of 917 adults were included for the final analysis.

### Exposure and outcome variables

RDW and albumin levels were measured as part of the routine laboratory assessments during hospital admission. RDW, expressed as a percentage, reflects the variability in the size of red blood cells and is typically measured by automated hematology analyzers. Serum albumin levels were measured using standard laboratory techniques. The RAR index was calculated by dividing the RDW value by the serum albumin level (%/g/L). The main outcomes were the occurrence of major adverse cardiovascular events (MACEs) during hospitalization, including 28-day in-hospital death, non-fatal stroke, non-fatal myocardial infarction, unplanned revascularization procedures, hospitalization for heart failure or angina.

### Covariates

After retrieved relevant articles and deeply evaluated, we selected the following confounding factors as covariates: age, sex (male or female), body mass index (BMI), systolic and diastolic blood pressure (SBP and DBP), fasting plasma glucose (FPG), total cholesterol (TC), triglycerides (TG), total bilirubin, high-density lipoprotein cholesterol (HDL-C), low-density lipoprotein cholesterol (LDL-C), serum creatinine (SCr) and glycated hemoglobin (HbA1c). Hypertension was defined in patients with systolic blood pressure (SBP) ≥ 140 mm Hg and/or diastolic blood pressure (DBP) ≥ 90 mm Hg, or currently using anti-hypertensive medications. Diabetes was defined as examination of FBG ≥7 mmol/L, a HbA1c ≥ 6.5%, or currently using anti-diabetic medications.

### Statistical analyses

Patients were divided into four groups based on quartiles of RAR index [grouped into quartiles: Q1 (reference), Q2, Q3, Q4]. Chi^2^-test and ANOVA tests were used to test for difference between the four groups. The characteristics of the patients were expressed by the mean and standard deviation (SD) for continuous variables, or frequencies (%) for categorical variables. Multivariable logistic regression analysis was used to calculate their odd ratio (OR) and corresponding confidence interval (95% CI) with adjustments that assessed the relationship RAR index of with MACEs among individuals with AMI. Outcomes were presented as Model 1 (unadjusted), Model 2 (adjusted for age, sex, BMI), Model 3 [adjusted for age, sex, BMI, HbA1c, estimated glomerular filtration rate (eGFR) (5), SBP, DBP, TC, TG, FBG, total bilirubin]. Restricted cubic spline (RCS) models were used to assess the dose-response relationship between RAR index and the incidence of MACEs. In addition, receiver operating characteristic curve (ROC) analysis was fitted to assess the accuracy of RAR index in predicting MACEs. Subgroup analyses were conducted stratified by age (age <60 and age ≥60), sex (male and female), hypertension (no and yes), AMI types (STEMI and NSTEMI), diabetes groups (no and yes).

All analyses were performed using R software version 4.4.3 (http://www.R-project.org). A two-tailed *P* values of < 0.05 was considered as statistically significant. The visualization of the results was created using the “ggplot2” package. The dose-response relationship between RAR index and the incidence of MACEs was calculated using the “RCS” package.

## Results

### Baseline characteristics

A total of 917 patients with AMI were included in this study. The mean age of the patients was 63.3 ± 13.4 years, with 75.7% of the patients being male. The patients were divided into four groups across the four quartiles of RAR index, and the demographic characteristics of the four groups are shown in Table 1. There were significant differences among the four groups of patients in terms of age, sex, BMI, total cholesterol, triglyceride, eGFR, SBP, DBP, hypertension, MACEs. It was worth noting that the high RAR index group exhibited a higher average age and an increased incidence of of MACEs, but presented lower levels of BMI, total cholesterol, triglyceride, eGFR, SBP, DBP, hypertension.

**Table 1:** Baseline characteristics of included participants.

Characteristics	BAR index				
	Q1 (≤ 3.14)	Q2 (3.15–3.47)	Q3 (3.48–3.90)	Q4 (>3.90)	*P* value
Participants, no (%)	227 (24.8)	227 (24.8)	234 (25.4)	229 (25.0)	
Age (years)	54.9 ± 12.0	61.8 ± 12.5	65.6 ± 12.4	70.8 ± 11.6	< 0.001
Sex (%)					< 0.001
Male	196 (86.3)	175 (77.1)	168 (71.8)	155 (67.7)	
Female	31 (13.7)	52 (22.9)	66 (28.2)	74 (32.3)	
BMI (kg/m^2^)	25.2 ± 1.5	24.9 ± 1.7	24.9 ± 1.5	24.8 ± 1.4	0.033
Total cholesterol (mmol/L)	4.8 ± 1.1	4.8 ± 1.3	4.7 ± 1.2	4.5 ± 1.3	0.026
Triglyceride (mmol/L)	1.9 ± 1.6	1.6 ± 1.4	1.5 ± 1.1	1.2 ± 0.8	< 0.001
Fasting glucose (mg/dl)	7.3 ± 2.2	7.1 ± 2.4	7.1 ± 2.5	7.6 ± 3.4	0.101
Total Bilirubin (umol/L)	14.4 ± 7.4	13.9 ± 7.4	14.6 ± 9.9	14.3 ± 9.7	0.872
HbA1c, (%)	6.6 ± 1.3	6.5 ± 1.1	6.5 ± 1.1	6.6 ± 1.5	0.322
HDL (mmol/L)	1.2 ± 0.4	1.2 ± 0.3	1.2 ± 0.3	1.2 ± 0.3	0.801
LDL (mmol/L)	2.9 ± 1.0	3.0 ± 1.0	2.9 ± 1.2	2.8 ± 1.0	0.368
eGFR (ml/min/1.73 m^2^)	98.3 ± 31.7	91.9 ± 31.9	88.4 ± 32.1	78.2 ± 42.9	< 0.001
SBP (mmHg)	133.4 ± 22.0	130.9 ± 20.3	130.0 ± 21.4	127.6 ± 21.6	0.031
DBP (mmHg)	84.5 ± 16.0	81.3 ± 13.2	79.9 ± 13.3	76.9 ± 14.4	< 0.001
RDW	12.6 ± 0.6	13.0 ± 0.7	13.3 ± 0.8	14.0 ± 1.2	< 0.001
Albumin (g/L)	43.4 ± 2.9	39.2 ± 2.1	36.3 ± 2.3	31.4 ± 3.6	< 0.001
Hypertension (%)	116 (51.1)	89 (39.2)	95 (40.6)	78 (34.1)	0.002
Diabetes mellitus (%)	161 (70.9)	171 (75.3)	175 (74.8)	176 (76.9)	0.515
MACEs (%)	22 (9.7)	31 (13.7)	46 (19.7)	57 (24.9)	< 0.001

Data are presented as the mean ± SD or number (%), as appropriate. BMI, body mass index; HbA1c, glycated hemoglobin; HDL, high-density lipoprotein; LDL, lowdensity lipoprotein; eGFR, estimated glomerular filtration rate; RDW, red blood cell distribution width; RAR, red blood cell distribution width to albumin ratio; SBP, systolic blood pressure; DBP, diastolic blood pressure; MACEs, major adverse cardiovascular events.

### The association of RAR index with MACEs in all participants

Table 2 showed the effects of RAR index on risk of MACEs in patients with AMI. In primary model (Model 1), one unit increase in RAR index was found to be signifi cantly associated with an increased occurrence of MACEs [OR = 1.55 (1.22, 1.97); *P* < 0.001]. In Model 2, after adjusting for age, sex, and BMI, the RAR index exhibited a significantly positive association with incidence of MACEs [OR = 1.36 (1.04, 1.76); *P* = 0.022]. In Model 3, following further adjustment for glycated hemoglobin, estimated glomerular filtration rate, systolic blood pressure, diastolic blood pressure, total cholesterol, triglyceride, fasting glucose, total bilirubin, the positive association between RAR index and MACEs was still remained [OR = 1.34 (1.03, 1.74); *P* = 0.030]. Comparison was also performed using quartiles of RAR index. The 1st quartiles of RAR index were used as a reference, and a significantly higher possibility of MACEs was found in 3st, 4st quartiles, regardless of the model type (All *P* for trend <0.001). Model 3 revealed a 2.30-fold likelihood risk for patients with the highest RAR index than for those with the lowest RAR index [Q3 vs. Q1: OR = 1.86 [1.05, 3.28], *P* = 0.034; Q4 vs. Q1: OR = 2.30 [1.28, 4.12], *P* = 0.005].

**Table 2:** Hr (95% CIs) for mortality according to the BAR index.

Characteristic	Model 1		Model 2		Model 3	
	OR (95% CI)	*P* value	OR (95% CI)	*P* value	OR (95% CI)	*P* value
MACEs						
BAR index (continous)	**1.55** (**1.22–1.97)**	**<0**.**001**	**1.36** (**1.04–1.76)**	**0**.**022**	**1.34** (**1.03–1.74)**	**0**.**030**
BAR index (category)						
Q1	Ref		Ref		Ref	
Q2	1.47 (0.82–2.63)	0.190	1.31 (0.72–2.36)	0.377	1.29 (0.71–2.33)	0.402
Q3	**2.28** (**1.32–3.93)**	**0**.**003**	**1.89** (**1.07–3.34)**	**0**.**028**	**1.86** (**1.05–3.28)**	**0**.**034**
Q4	**3.09** (**1.81–5.26)**	**<0**.**001**	**2.36** (**1.32–4.22)**	**0**.**004**	**2.30** (**1.28–4.12)**	**0**.**005**
*P* for trend		<0.001		0.034		0.032

OR, odd ratio; CI, confidence interval; BAR, red blood cell distribution width to albumin ratio. Model 1: unadjusted. Model 2: adjusted for age, sex, and BMI. Model 3: adjusted for age, sex, body mass index, glycated hemoglobin, estimated glomerular filtration rate, systolic blood pressure, diastolic blood pressure, total cholesterol, triglyceride, fasting glucose, total bilirubin.

*P*<0.05 is marked in bold type.

### ROC curve analysis of the value of the RAR index

**Figure 1:**
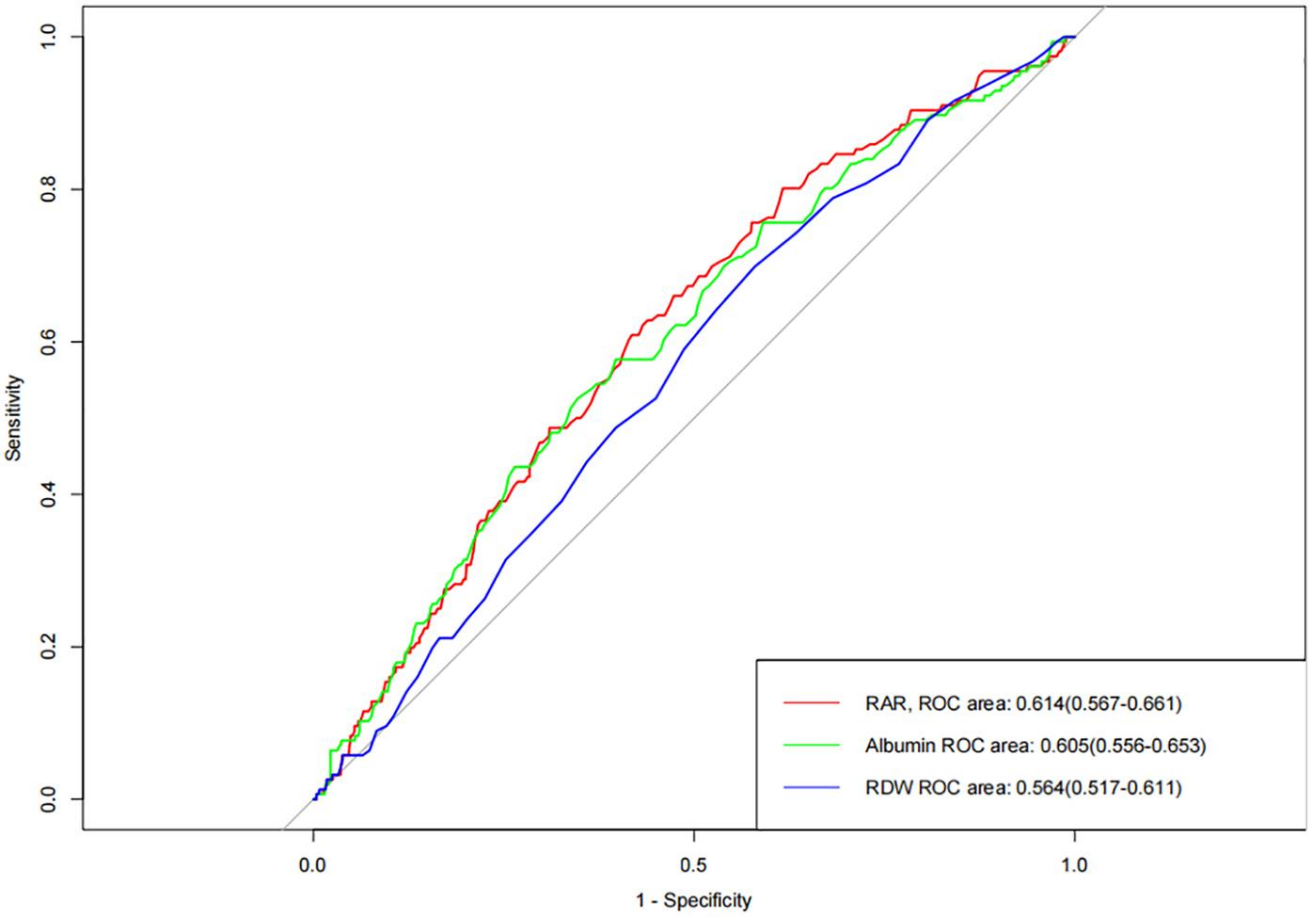
Receiver operating characteristic curve analysis of the accuracy of RAR index in predicting the occurrence of MACEs.

Figure 1 presents the analysis results of the ROC curve, which aims to evaluate the predictive value of RDW, albumin, and RAR index for the occurrence of MACEs in patients with AMI. The results showed that when RDW and albumin were used to predict the occurrence of MACEs in AMI patients, the area under the curve (AUC) of RDW and albumin was 0.564 and 0.605, suggesting that these two indicators have a certain predictive ability for the occurrence of MACEs. Further analysis indicates that RAR index, as the composite indicator constructed by RDW and albumin, shows a slight upward trend in AUC compared to a single indicator, with the AUC of 0.614.

### The non-linear relationship between RAR index and the likelihood of MACEs

**Figure 2:**
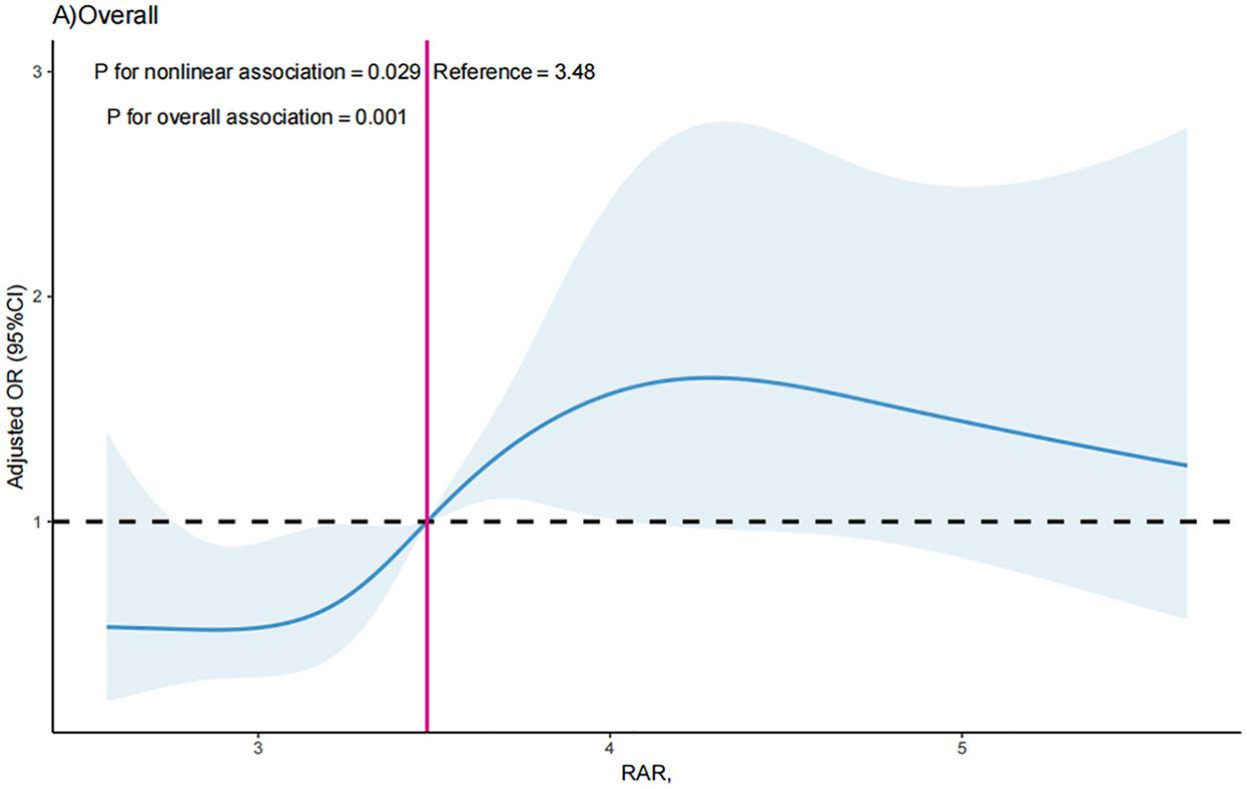
The dose-response relationship between RAR index in predicting the occurrence of MACEs.

The RCS curve was conducted in to visualize the association between RAR index with MACEs in all participants after adjustment for all the tested confounders, and the results showed that RAR index was shown to have a nonlinear correlation with likelihood of MACEs (*P* for nonlinearity = 0.029). The critical value of the RAR index for predicting MACEs was 3.48 (Figure 2). The RCS analysis revealed a S-shaped association between RAR index and possibility of MACEs, and when the RAR index surpassed 3.48, the OR of MACEs increased significantly as the RAR index increased [OR = 2.17 (1.11, 3.25); *P* = 0.031]. For RAR index ≤3.48, each one unit increases in RAR index did not correlate with increased likelihood of MACEs [OR = 1.08 (0.75, 1.55); *P* = 0.693].

### Subgroup analysis

**Figure 3:**
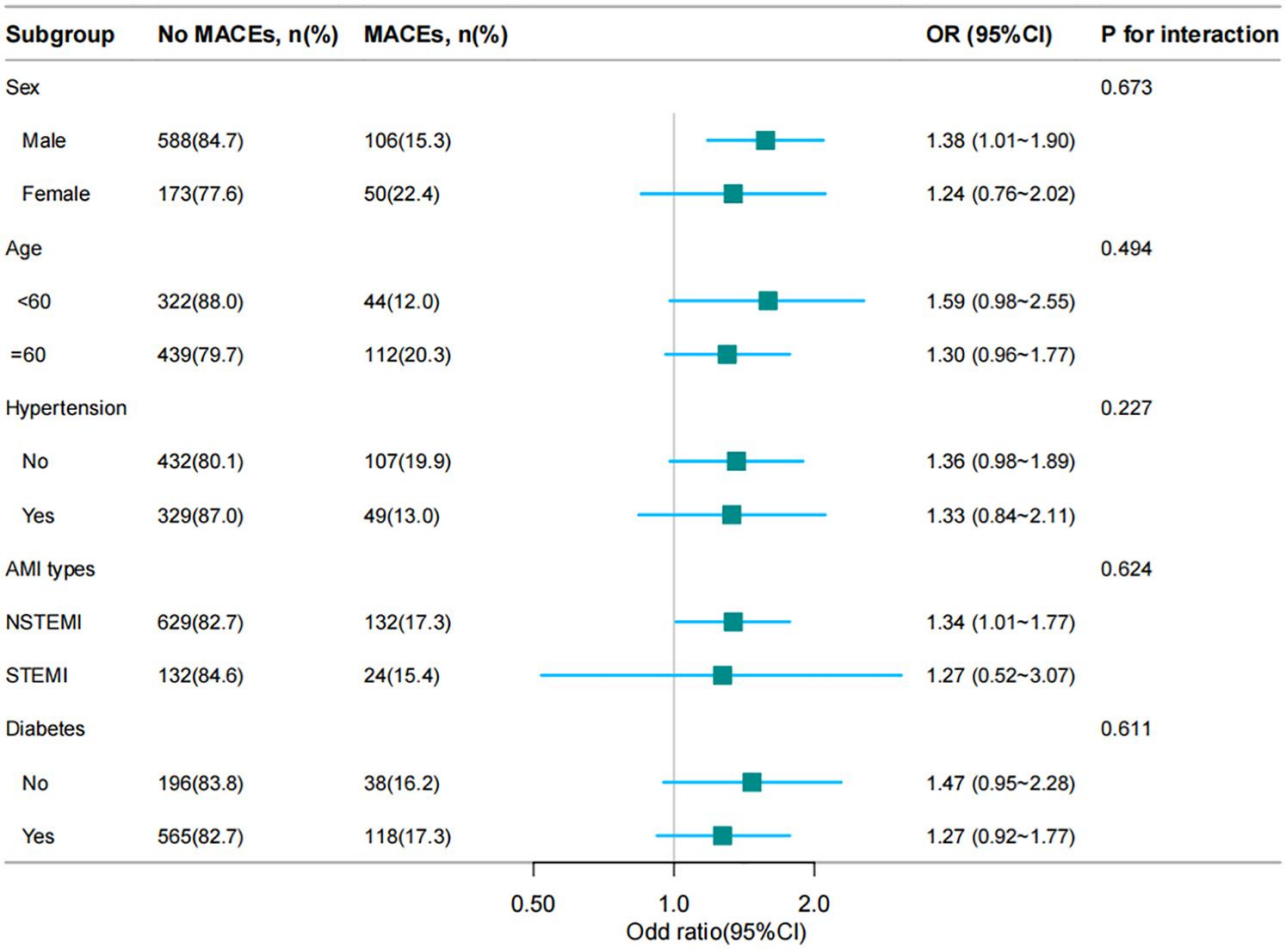
Forest plot of stratified analysis of the potential impact of confounders.

To discover the association between the RAR index and the incidence of MACEs and whether it was potentially influenced by confounding factors, stratified analyses were conducted on age, sex, STEMI, hypertension and diabetes. The results were displayed as Figure 3, which suggested this association varied by sex and STEMI. The RAR index was positively associated with the incidence of MACEs [OR = 1.57 (1.18, 2.09); *P* *=* 0.002] in male patients. Moreover, the RAR index exhibited significant association with occurrence of MACEs in STEMI group [OR = 1.32 (1.01, 1.77); *P* *=* 0.045].

**Figure 4:**
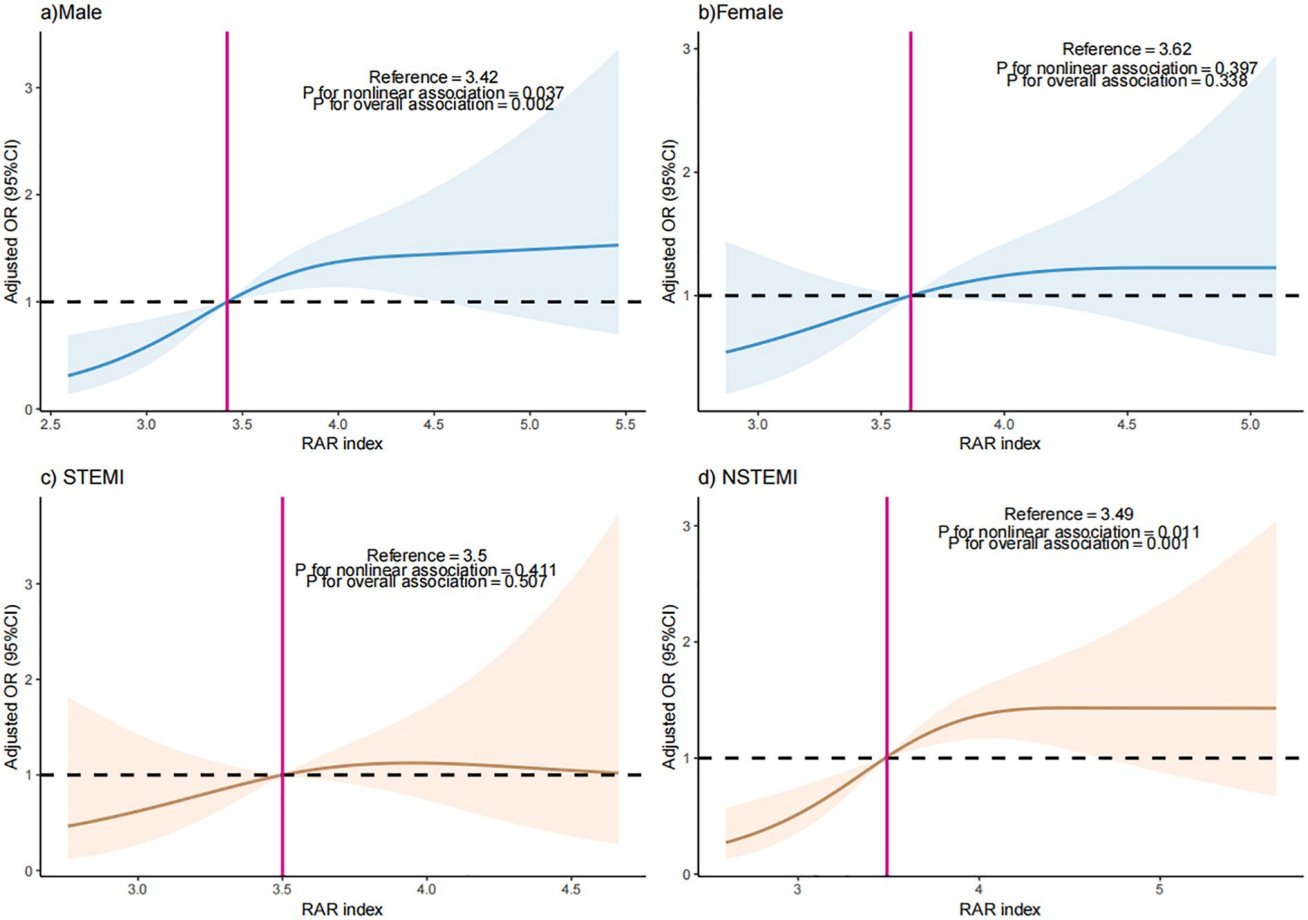
The dose-response relationship between RAR index and the occurrence of MACEs in **(a)** male patients, **(b)** female patients, **(c)** STEMI patients, and **(d)** STEMI patients.

Participants were separated as “male” and “female” by sex, or “Yes” and “No” by STEMI. The RAR index was also included as a continuous variable in RCS analysis. Results of RCS showed a nonlinear relationship between RAR index and the occurrence of MACEs (*P* for nonlinearity = 0.037) in the male participants. A nonlinear correlation was also found in NSTEMI participants (*P* for nonlinearity = 0.011) (Figure 4).

## Discussion

As far as we know, this is the first report to explore the association between RAR index and the incidence of MACEs in patients with AMI. Conclusively, the RAR index exhibited a nonlinear positive relationship with incidence of MACEs among individuals with AMI, and this association varied by sex and AMI types. Specifically, the inverted J-shaped relationship exists between the RAR index and the possibility of MACEs in female patients and STEMI patients. The incidence of MACEs demonstrates a significant increase in all patients when the RAR exceeds 3.48. Notably, in male patients and patients with STEMI, the occurrence of MACEs significantly escalates when the RAR index surpasses 3.42 and 3.49, respectively.

### Individual prognostic significance of RDW and albumin

RDW reflects the heterogeneity of red blood cell volume, which is closely linked to inflammation and oxidative stress. Lippi et al. demonstrated that RDW is significantly positively correlated with inflammatory markers (e.g., TNF-α, IL-6) in patients with cardiovascular diseases, confirming its role as a surrogate for systemic inflammation (6). These inflammatory factors can interfere with the erythropoiesis process, leading to an increase in the heterogeneity of red blood cell size and subsequently raising RDW. For instance, a study pointed out that in people with cardiovascular diseases, the inflammatory state shows a significant positive correlation with the increase of RDW (7). Meanwhile, the large number of reactive oxygen species (ROS) produced by oxidative stress can damage the red blood cell membrane, affecting its normal function and morphology, and also have an impact on RDW (8, 9). In our cohort, elevated RDW was independently associated with poorer survival outcomes (HR = 1.23, 95% CI: 1.08–1.40, *p* = 0.002), aligning with findings in gastrointestinal cancers where RDW elevation reflects impaired iron metabolism and chronic inflammation, both of which accelerate tumor progression (10).

Serum albumin, as the most abundant protein in plasma, not only plays a crucial role in maintaining colloid osmotic pressure but also possesses antioxidant and anti-inflammatory properties (11, 12). When AMI occurs in the body, inflammatory responses and tissue damage can lead to a reduction in albumin synthesis and an increase in catabolism, resulting in a decrease in serum albumin levels. This change in albumin level, together with the alteration of RDW, constitutes the fluctuation of the RAR index, thereby affecting the risk of MACEs occurrence (13). Our study demonstrated that lower albumin was a strong standalone predictor of adverse prognosis (HR = 1.56, 95% CI: 1.32–1.85, *p* < 0.001), consistent with its role in maintaining vascular integrity and modulating the inflammatory response (14).

### Prognostic value of RAR in previous studies

Compared with the single indicator of RDW, composite indicators combining it with other indicators have greater superiority in predicting disease outcomes. For example, the combination of RDW and platelet ratio has shown significant predictive value in AMI patients (15). Jian et al. reported that RAR had a higher AUC (0.738) than RDW alone (0.624) or albumin alone (0.696) in predicting short-term prognosis in AMI patients, consistent with our finding that RAR (AUC = 0.614) outperforms individual indicators (16). Li et al. further demonstrated that elevated RAR was significantly associated with increased 30-day mortality in AMI patients, with a stronger predictive power than either component (17). Similarly, Li and Xu confirmed that RAR independently predicts adverse outcomes in AMI, emphasizing its potential for risk stratification (18). RAR index seems to be more sensitively reflect the body's inflammatory state and pathophysiological disorders. If the RAR index can be verified in larger-scale prospective studies, it is expected to become a simple, economical and effective biomarker for risk stratification of AMI patients.

The influence of gender differences on the relationship between RAR index and MACEs is also worthy of in-depth exploration. In female patients, the presence of estrogen is an important influencing factor. In female patients, estrogen may exert cardiovascular protective effects by improving endothelial function, inhibiting platelet aggregation, and reducing inflammation (19). Premenopausal women, with higher estrogen levels, generally have lower cardiovascular risk, but this protection diminishes postmenopause, aligning with our subgroup analysis showing weaker associations in females (20). In contrast, male patients may exhibit stronger associations due to higher rates of adverse lifestyle factors (e.g., smoking, excessive alcohol consumption), which exacerbate inflammation and vascular damage (21, 22), potentially amplifying the RAR-MACEs link.

Variations in AMI subtypes also affect the RAR-MACEs relationship. STEMI involves acute complete coronary occlusion, triggering intense inflammatory responses and extensive myocardial damage (23, 24), which may enhance RAR's predictive sensitivity. This is supported by our finding that RAR was significantly associated with MACEs in STEMI patients. In contrast, NSTEMI involves partial occlusion and milder inflammation (25, 26), which may explain the weaker, albeit nonlinear, association observed in this subgroup. Therefore, the variation characteristics of the RAR index in these two types of patients may differ from its predictive value for MACEs. This suggests that when clinicians evaluate AMI patients, they should interpret the significance of the RAR index differently based on different AMI types.

### Limitations

However, this study has certain limitations. Firstly, as a retrospective study, its data collection may be affected by hospital case selection bias, and the included patients may not fully represent the entire AMI patient population, leading to limited extrapolation of the study results. Secondly, the RAR index is a composite indicator. Although this study found an association between it and MACEs, it is currently unclear to what extent RDW and albumin contribute to this association respectively. In addition, some potential confounding factors may not have been fully considered in the study, such as the genetic background of patients, the long-term use of certain drugs (such as antiarrhythmic drugs, antihypertensive drugs, etc.), and the influence of other comorbidities (such as chronic obstructive pulmonary disease, chronic kidney disease, etc.) on the relationship between the RAR index and MACEs.

Future research should focus on conducting multi-center, large-sample prospective cohort studies to more extensively verify the prognostic value of the RAR index in AMI patients of different regions and races. Meanwhile, in-depth mechanism research should be carried out. Through cell experiments, animal experiments and other means, the specific mechanism of action of RDW and albumin in influencing the occurrence of MACEs and the interrelationship between them should be clarified. Attention should also be paid to the changing characteristics and clinical significance of the RAR index in gender, AMI types, and the risk assessment system should be further refined to improve the overall diagnosis and treatment level for AMI patients.

## Conclusion

This study revealed that the RAR index is an independent predictor of MACEs in AMI patients, with a non-linear relationship and a critical threshold of 3.48. Its predictive performance surpasses that of individual RDW or albumin, and its clinical significance varies by sex and AMI sub-type. These findings suggest that the RAR index could serve as a simple, cost-effective biomarker for risk stratification and prognosis assessment in AMI patients, warranting further validation in prospective studies.

## Data Availability

The raw data supporting the conclusions of this article will be made available by the authors, without undue reservation.
